# Reproducible Clusters from Microarray Research: Whither?

**DOI:** 10.1186/1471-2105-6-S2-S10

**Published:** 2005-07-15

**Authors:** Nikhil R Garge, Grier P Page, Alan P Sprague, Bernard S Gorman, David B Allison

**Affiliations:** 1 Department of Biostatistics, Section of Statistical Genetics, Ryals Public Health Building Suite 327, University of Alabama at Birmingham, Birmingham, USA; 2 Computer and Information Sciences department, University of Alabama at Birmingham, Birmingham, USA; 3 Department of Psychology, Hauser Hall, Hofstra University, NY 11550, USA; 4 Medical College of Georgia, 1120 15th Street, Ca4100, Augusta, GA 30912, USA. email: ngarge@mcg.edu

## Abstract

**Motivation:**

In cluster analysis, the validity of specific solutions, algorithms, and procedures present significant challenges because there is no null hypothesis to test and no 'right answer'. It has been noted that a replicable classification is not necessarily a useful one, but a useful one that characterizes some aspect of the population must be replicable. By replicable we mean reproducible across multiple samplings from the same population. Methodologists have suggested that the validity of clustering methods should be based on classifications that yield reproducible findings beyond chance levels. We used this approach to determine the performance of commonly used clustering algorithms and the degree of replicability achieved using several microarray datasets.

**Methods:**

We considered four commonly used iterative partitioning algorithms (Self Organizing Maps (SOM), K-means, Clutsering LARge Applications (CLARA), and Fuzzy C-means) and evaluated their performances on 37 microarray datasets, with sample sizes ranging from 12 to 172. We assessed reproducibility of the clustering algorithm by measuring the strength of relationship between clustering outputs of subsamples of 37 datasets. Cluster stability was quantified using Cramer's *v*^*2 *^from a *kXk *table. Cramer's *v*^*2 *^is equivalent to the squared canonical correlation coefficient between two sets of nominal variables. Potential scores range from 0 to 1, with 1 denoting perfect reproducibility.

**Results:**

All four clustering routines show increased stability with larger sample sizes. K-means and SOM showed a gradual increase in stability with increasing sample size. CLARA and Fuzzy C-means, however, yielded low stability scores until sample sizes approached 30 and then gradually increased thereafter. Average stability never exceeded 0.55 for the four clustering routines, even at a sample size of 50. These findings suggest several plausible scenarios: (1) microarray datasets lack natural clustering structure thereby producing low stability scores on all four methods; (2) the algorithms studied do not produce reliable results and/or (3) sample sizes typically used in microarray research may be too small to support derivation of reliable clustering results. Further research should be directed towards evaluating stability performances of more clustering algorithms on more datasets specially having larger sample sizes with larger numbers of clusters considered.

## Introduction

Cluster analysis is a statistical approach used in microarray research that identifies genes within a cluster that are more similar to each other than genes contained in different clusters. By grouping genes that exhibit similarities in their expression patterns, the function of those genes which were previously unknown may be revealed. There are two groups of clustering methods, hierarchical and non-hierarchical. Non-hierarchical algorithms require the number of clusters (k) be pre-specified. Non-hierarchical algorithms can run multiple times with different values of k. The user can then choose the clustering solution that is logical to address the problem of interest.

If we consider each gene as a point in high dimensional space, then *"clusters may be described as continuous regions of this space containing a relatively high density of points, separated from other such regions by regions containing a relatively low density of points. Clusters described in this way are sometime referred to as natural clusters" *[1].

Despite the use of cluster analysis in microarray research, the evaluation of the "validity" of a cluster solution has been challenging. This is due, in part, to the properties of cluster analysis. Cluster analysis has no null hypothesis to test and hence no right answer, which makes the testing of the validity of specific solutions, algorithms, and procedures difficult [2]. A second challenge encountered is that genes may not "naturally" fall into clusters separated by empty areas of the attribute space in genome expression studies. Hence, genome-wide collections of expression trajectories may lack a "natural clustering" structure in many cases [1]. Third, the result of gene clustering may be "method sensitive". That is, gene clustering depends on several methodological choices, including the distance metric used, the clustering algorithm, and the stopping rule in the case of iterative partitioning methods. Hence, it is important to evaluate the stability of any specific derived cluster solution and the general performance of clustering approaches.

According to McShane et al., *"Clustering algorithms always detect clusters, even in random data and it is imperative to conduct some statistical assessments of the strength of evidence for any clustering and to examine the reproducibility of individual clusters" *[3]. Roth et al. defined stability as *"the variability of solutions which are computed from different data sets sampled on the same source" *[4]. It has been noted that a replicable classification is not necessarily a useful one, but a useful one that characterizes some aspect of the population must be replicable [5]. The concept of a replicable cluster is defined as reproducible across multiple samplings from the same population. Thus, some methodologists have suggested that the validity of clustering methods could be defined as the extent by which they yield classifications that are reproducible beyond chance levels. Most recently, Tseng *et al. *[6] identified stability of clusters in a sequential manner through an analysis of the tendency of genes to be grouped together under repeated resampling. Famili *et al. *[7] summarized the related work as follows:

*Zhang et al*. [8]*proposed a parametric bootstrap re-sampling method (PBR) to incorporate information on variations in gene expression levels to assess the reliability of gene clusters identified from large-scale gene expression data...Smolkin et al*. [9]*assessed the stability of a cluster using their Cluster Stability Score, by which a cluster's stability is calculated through clustering on random subspace of the attribute space...Ben-Hur et al*. [10]*proposed a stability-based re-sampling method for estimating the number of clusters, where stability is characterized by the distribution of pair-wise similarities between clusters obtained from sub-samples of the data...Datta et al*. [11]*formulated 3 other validation measures using the left-out-one condition strategy to evaluate the performances of 6 clustering algorithms...Giurcaneanu et al*. [12]*introduced a stability index to estimate the quality of clusters for randomly selected subsets of the data*.

Clusters that produce classifications with greater replicability would be considered more valid [5]. The objective of this paper is to determine the performance of commonly used non-hierarchical clustering algorithms and the degree of stability achieved using several microarray datasets.

## Methods

### Data

#### Real datasets

We considered 37 real microarray datasets of various kinds and from various sources (See Table 1). Most of these microarray datasets were downloaded from Gene Expression Omnibus (GEO) [13] – a public repository of microarray datasets and few from other sources listed in Table 1. We evaluated their stability performances on various non-hierarchical clustering algorithms. We included datasets containing different experimental designs, such as (1) time series: – samples under a particular condition observed at various time points, (2) cross sectional: – subsets of samples under various conditions, and (3) case-control experiments: – i.e., case samples (having the problem/disease) and control samples (not having the problem/disease). These data are drawn from a variety of species, tissue types, and laboratories.

#### Simulated datasets

We also evaluated stability performances on simulated datasets for two major reasons: a) to validate our methodology of stability computation and b) to observe the stability behaviour with very large sample sizes which were not available in real datasets. We simulated 8 datasets with 1200 genes and sample sizes ranging from n = 20 to 1000, where n is the number of subjects. All simulated datasets were structured for 6 clusters (k = 6) with correlation *ρ *set to (0.33)^1/2 ^for all pairwise combinations of genes within clusters and zero for all pair wise combinations of genes in different clusters. In order to validate our methodology, we would predict higher scores when we extract 6 clusters in our fitted solutions. Simulated datasets also help us understand the stability behaviour for values other than k = 6 (i.e., when we extract the wrong number of clusters). Table 2 explains the details of simulated datasets. We acknowledge that number of genes in simulated datasets is smaller than real datasets. At larger sample sizes (n = 250, 500, and 1000), simulating more genes, producing clustering results and computing stability becomes computationally prohibitive. The main purpose of simulating datasets is to validate our methodology *i.e. *to check if we get correct scores for the right number of clusters (k = 6 in our case). For this purpose, 1200 genes suffice.

#### Preprocessing of data

Microarray datasets may contain unobserved expression levels termed, i.e., missing values. The first stage of our preprocessing handled these missing values and then a second stage standardized the variables to mean zero and unit variance as explained below.

#### Missing values

If we represent microarray data as a matrix with rows representing genes and columns representing chips or samples, we filtered out all rows which contained at least one null expression or missing value because we do not know the exact source(s) for the missing/null value observation. Missing data can be due to array damage, transcription errors, etc. Conventional algorithms for clustering require complete datasets to run and extending these clustering routines to accommodate missing data was beyond the scope of our inquiry.

#### Standardization

Variables such as gene expression values measured on different scales can affect cluster analysis [14]. The main purpose of standardization is to convert variables measured on different scales to a unitless standard scale. One might question the reason to standardize genes when microarray dataset represents expression levels of various genes. But a level of mRNA (messenger ribonucleic acid) expression (for a given gene) responsible for triggering specific biological activity can be different for different genes. Therefore each gene vector (expression values of a gene across samples) may be a measurement made on a different functional scale. To address this issue, we standardized each gene vector (expression values of a gene across samples) and replaced expression values by Z scores before clustering genes. Z scores were computed using the following formula [15-17]:



Where Z_ij _= Z score computed for expression level observed for gene i in sample/subject j,  = intensity measured for gene i in sample j, and  = mean intensity of gene i across samples,  = standard deviation of expressions of gene i across samples.

### Clustering methods

There exist many clustering algorithms which take microarray datasets as input and produce clusters as output. Some algorithms, particularly non-hierarchical algorithms, require that the number of clusters (k) be pre-specified, whereas others do not. Those that require k as an input parameter can be run multiple times with different values of k. The user can then choose the clustering solution that seems best to address the problem of interest. Our research suggests a statistical criterion for selecting the right number of clusters by quantifying stability scores using Cramer's *v*^*2 *^from *kXk *contingency table. Since this criterion takes the number of clusters (k) into account, we restrict our attention to iterative partitioning clustering methods. Most iterative partitioning methods function in the following manner [5]:

1. Begin with an initial partitioning of the dataset into a specified number of clusters (k) and thereafter compute the centroids of these clusters.

2. Allocate each data point to the cluster that has the nearest centroid (except Fuzzy C-means where data points belong to a cluster that is specified by a membership grade).

3. Compute the new centroids of the clusters. Clusters are not updated until there has been a complete pass through the data.

4. Alternate steps 2 and 3 until no data points change clusters.

We consider the following four iterative partitioning methods, which are commonly used in the literature. The algorithms for them are freely available in R statistical package.

#### K-means

In K-means clustering, one decides on the number of clusters and randomly assigns each gene to one of the *k *clusters. If a gene is actually closer to the center of another cluster, as assessed by variety of similarity metrics (i.e., Pearson's correlation or Euclidean Distance) the gene will be assigned to the closer cluster. After assigning all genes to the closest cluster, the centroids (centers of clusters) are recalculated. After a number of iterations, the cluster centroids will no longer change and the algorithm stops. The K-means clustering is described in detail in [18]. However, the efficient version of the algorithm is presented by Hartigan and Wong [19] which is implemented in R (publicly available software). This version of K-means assumes that it is not practical to require that the solution has minimal sum of squares against all partitions, except when *M *(number of genes to be clustered), *N *(number of chips or samples) are small and *k *= 2. For details of this algorithm, please refer [19].

#### Self Organizing Map (SOM)

Self Organizing Map (SOM) is a clustering algorithm [20] used to map high dimensional microarray data onto a two-dimensional surface. It is similar to K-means, but instead of allowing of centroids to move freely in high dimensional space, they are restricted to a two-dimensional grid. Grid maps considered by us are *1 × 2, 1 × 3, 1 × 4, 1 × 5, 1 × 6, 1 × 7, 1 × 8, 1 × 9, 1 × 10 *for k = 2 to 10 respectively. We did not assess stability for other grid structures to see if we obtain similar stability scores, because assessing stability on 37 datasets with different set of grid structures for k = 2 to k = 10 involves impractical computations. The grid structure implies a relationship between neighboring clusters on the grid. The resultant map is organized in such a way that similar genes are mapped onto similar clusters (nodes) or to neighboring clusters. Hence, the arrangement of clusters reflects the topological relationships of these clusters.

#### Clustering LARge Applications (CLARA)

The clustering algorithm PAM (Partition Around Medoids) works effectively for small datasets but does not scale well for large datasets [21]. To deal with large datasets, a *sampling*-based method, called CLARA (Clustering LARge Applications) can be used. CLARA [22] is carried out in two steps. First it draws a sample of dataset, applies PAM algorithm on the sample and finds *k representative objects *of the sample. In PAM, one considers possible choices of *k representative objects *and then constructs the clusters around these representative objects. A set of *k representative objects *is selected which gives minimum average dissimilarity. PAM algorithm is explained in detailed in [23].

Once the *k representative objects *are selected, then each object not belonging to the sample is assigned to the nearest of the k representative objects. This yields clustering of the entire dataset and measure of quality of this clustering is obtained by computing the average distance between each object of the dataset and its representative object. After five samples have been drawn and clustered, the one is selected for which the lowest average distance was obtained.

#### Fuzzy C-means

Fuzzy C-means is a data clustering technique wherein each gene belongs to a cluster that is specified by a membership degree. Membership degrees between zero and one are used instead of crisp assignments of the data to clusters. This technique was originally introduced by Bezdek [24]. In our methodology we use crisp assignments of genes to clusters. Hence, in Fuzzy C-means we assign every gene to a unique cluster – the one showing maximum degree of membership for that gene. One may question why K-means is considered different from Fuzzy C-means if we do not assign genes to more than one cluster in Fuzzy C-means? In K-means [19], an early assignment to a given cluster may preclude a gene from being considered to any other cluster. Crisp assignment (in K-means algorithm) may prematurely force a gene into a cluster. Fuzzy C-means on other hand can be considered more "global" where a gene is assigned to more than one cluster with some membership degree (0 to 1) and then we convert the fuzzy membership into crisp membership by assigning the gene to a cluster showing maximum degree of membership. The above two approaches may produce different clustering solutions and hence Fuzzy C-means without fuzziness is not same as K-means.

### Similarity Metric

The similarity metric allows us to compute the distance between two objects to be clustered. Two of the more common similarity metrics are: Pearson's correlation coefficient and Euclidean distance. A correlation coefficient evaluates the direction of change between two expression profiles. It is described as a shape measurement, which is insensitive to differences in magnitude of the variables. The value of correlation coefficient ranges from -1 to +1, and values of zero indicate a random relationship between profiles [5]. Euclidean distance is a dissimilarity measure, that is, a high distance implies low similarity and measures both magnitude and direction of change between two expression profiles. It can be shown that correlation and Euclidean distance are equivalent after standardization [16]. For our studies, we use Euclidean distance which can be calculated as:



Where, *d*_*ij *_is the distance between genes i and j (across N samples), and g_*ik *_is the gene expression value of the k^th ^sample/subject for the i^th ^gene.

Pearson's correlation coefficient can be defined as:



Where  is the mean intensity of gene g_*i *_across samples.

### Method used to compute cluster stability

We quantify stability/replicability using Cramer's *v*^*2 *^. Cramer's *v*^*2 *^makes use of *χ*^2 ^statistics. If we classify data by two systems simultaneously, the result is a two-way contingency table. One can analyze data of this type using the classic *χ*^2 ^test, an inferential test of the null hypothesis, which states there is no association between the two classification schemes (for details, refer [25]). One can also compute measures that quantify the degree of association in such tables [26]. One such measure, Cramer's *v*^*2 *^is the squared canonical correlation between two sets of nominal variables that define the rows and columns of the contingency table. It indicates the proportion of variance in one classification scheme that can be explained or predicted by the other classification scheme [25]. It ranges from 0 to 1, with 0 indicating no relationship and 1 indicating a perfect reproducibility.



Where *χ*^2 ^= is the ordinary *χ*^2 ^test statistic for independence in contingency tables [27], N = the number of items cross classified (i.e., total number of genes to be clustered), and k = the smaller of rows or columns in a two way contingency table, in our case, k is the number of clusters extracted.

## Algorithms and implementation

We implemented the algorithms explained in this section using R, a computer language designed for statistical data analysis. All four clustering techniques are implemented in R.

### Approach to compute cluster stability

**Figure 1:**
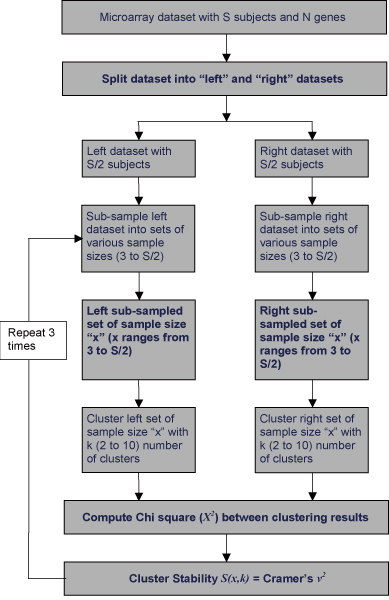
**Algorithm: cluster stability computation. **Cluster stability score *S(x,k) *is computed for every "k"(number of clusters) and every pair of sub-sampled set of sample size "x".

This approach is depicted in Figure 1. Let us assume that we have a microarray dataset with "S" subjects and "N" genes. We split this dataset into two halves – "left" and "right" datasets – each containing half (S/2) the number of subjects and N genes (algorithm for splitting the dataset is explained in detail below). We then resample the left dataset  times and create  samples. Each sample is created without replacement but it is replaced to create a next sample of higher sample size. For example, a sample of sample size 3 is created by randomly selecting 3 subjects without replacement from the left dataset. Then a new sample of sample size 4 is created by drawing (without replacement) one additional case/subject from those remaining in the left dataset. The above procedure is repeated  times each time adding in new case to the existing subsample to create  samples with sample sizes ranging from 3 to S/2 respectively. Similarly we resample from the right dataset and create  samples. Thereafter, all samples of the left dataset and right dataset are clustered with k number of clusters (k ranging from 2 to 10). We then generate *kXk *contingency tables for each pair of samples – one sample from left and another from the right dataset, both having same sample size x (x ranges from 3 to S/2). A cluster stability score *S(x,k) *is then quantified using Cramer's *v*^*2 *^for every *kXk *table. The random selection of subjects (columns of microarray datasets) to create samples may affect clustering solutions produced on those samples which, in turn, may produce stability scores by chance. As shown in Figure 1, this procedure is repeated thrice. Stability scores *S(x,k) *are computed thrice on each dataset and averaged to produce more reliable results.

### Algorithm to split dataset into two halves

**Figure 2:**
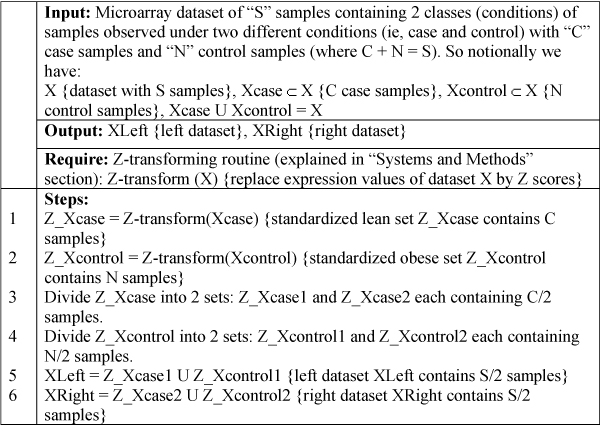
**Pseudo code of algorithm: Splitting dataset. **This algorithm explains steps involved in splitting a hypothetical dataset of sample size S containing samples observed under 2 conditions (say lean, obese) into left and right datasets.

A microarray dataset contains subjects observed under different conditions or time points. Blindly splitting a dataset into two halves may create "left" and "right" datasets that contain subjects observed under different conditions or contain unequal proportions of subjects observed under different conditions. Hence, in order to create "left" and "right" datasets containing same proportions of samples observed under different conditions we used the algorithm noted in the example contained in Figure 2. If we assume a dataset of "S" subjects observed under two different conditions (say case and control), then after applying this algorithm (Figure 2) we produce "left" and "right" datasets (each containing S/2 subjects) having same proportions of case to control subjects and expect a clustering algorithm to produce identical clustering solutions on both "left" and "right" datasets.

## Results

We evaluated stability performances on 37 real microarray datasets (Table 1) and 8 simulated datasets (Table 2).

### Results on real datasets

**Figure 3:**
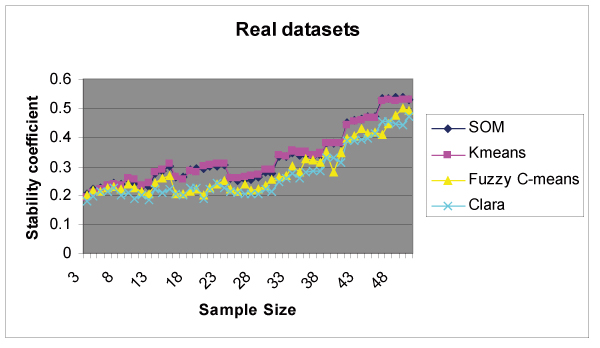
**Cluster Stability results. **Stability scores for various values of k (2 to 10) are computed on all 37 datasets. For each dataset, we selected a column (k) showing maximum summation of scores across sample size. Finally all 37 columns selected on 37 datasets were merged into one resultant column representing stability scores with respect to sample size for that clustering routine.

Stability results produced on a real dataset (n = 16, where n is number of subjects in dataset) with the SOM algorithm are shown in Table 3. Each cell of Table 3 represents the stability score computed for the value of k and the pair of samples. We produced 37 output tables for 37 real datasets of various sample sizes. Real datasets may have different cluster structures. Hence, for every output table produced on a given dataset, we selected a column k which gives a maximum summation of stability scores across sample sizes and consider it (k) as the best clustering structure for that dataset. We selected 37 columns of scores from 37 real datasets and merged them into one column by averaging scores across columns (k) for same sample sizes. The resultant column of scores represents the stability curve for that clustering algorithm across sample size. Figure 3 plots stability scores (summarized on 37 real datasets) with respect to sample size for all four clustering routines. All four methods showed increasing stability with increasing sample size. K-means and SOM showed a gradual increase in stability with increasing sample size. CLARA and Fuzzy C-means, however, maintained low stability scores until a sample size of 30 was attained. Stability scores then gradually increased after this threshold. K-means and SOM showed superior stability scores as compared to CLARA until the sample size attained n = 30. It is interesting to note that average stability achieved is not greater than 0.55 for all four clustering routines even when at sample size of n = 50 is attained. These results suggest that microarray datasets may lack natural clustering structure, thereby producing low stability scores on all four clustering methods. Alternatively, if we consider the 90^th ^percentile of scores across 37 selected columns (k) (37 columns of scores from 37 real datasets) for similar sample sizes to represent stability coefficients produced on datasets having clustering structure, we then observe scores between 0.7 and 0.8 until a sample size of n = 50 for the four clustering algorithms is achieved.

### Results on simulated datasets

**Figure 4:**
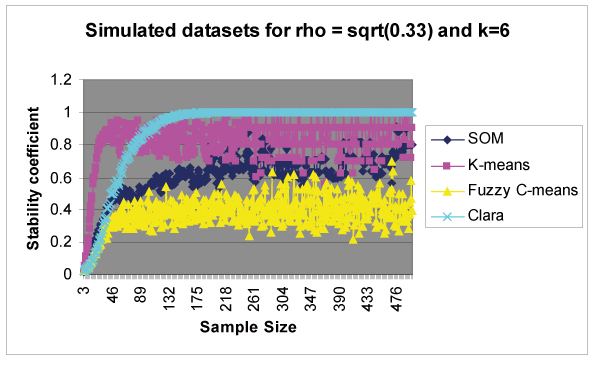
**Cluster Stability results on simulated datasets for k = 6. **Datasets are simulated with a clustering structure k = 6 (6 clusters). The above figure shows high stability scores observed for k = 6 on all four clustering routines.

**Figure 5:**
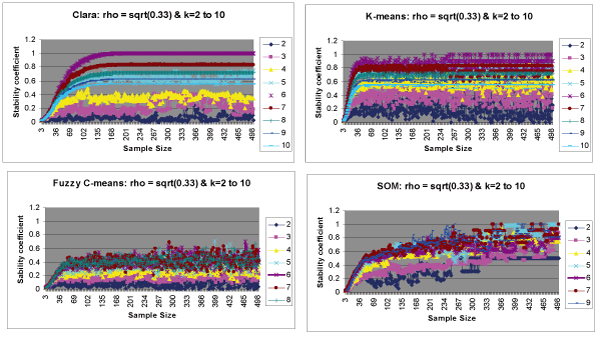
**Cluster Stability results on simulated datasets for k = 2 to k = 10. **Stability scores for various values of k (2 to 10) are computed on all the 8 simulated datasets. For each dataset, we generate an output table of scores (explained in Algorithms section). We merge all the 8 output tables produced into one table with each cell computed as average of corresponding cells in 8 tables. Finally scores are plotted for all k values with respect to sample size. For cleaner visualization purposes, we do not show stability curves for all k values in figure 5c and figure 5d. ***a ***Scores plotted for CLARA for each k (2–10). ***b ***Scores plotted for K-means for each k (2–10). ***c ***Scores plotted for Fuzzy Cmeans for each k (2–10). ***d ***Scores plotted for SOM for each k (2–10).

All 8 simulated datasets have the same clustering structure (k = 6) and the same correlation *ρ *set to (0.33)^1/2 ^within a cluster. Thus, (as expected) all datasets show high scores on k = 6 and low scores on other values of k. In simulated datasets, we merged all 8 output tables produced on 8 datasets into one output table with each cell computed as the mean of all the corresponding cells in 8 tables thereby producing the distribution of scores for each value of k (k ranging from 2 to 10) across sub-sampled space. The final output table manifests the stability behavior of the clustering algorithm for various values of clusters (k) considered. In simulated datasets, we produced a final output table of scores for each k (2 to 10) across sub-sampled space. We plotted stability results for various values of k across sample sizes as shown in Figure 5. As expected, maximal stability was achieved for the correct number of clusters k = 6 in all four clustering routines thereby validating our methodology and programming. However, as we deviate from k = 6, we observed a decline in stability scores. This phenomenon can be clearly observed in CLARA, K-means and Fuzzy C-means (Figure 5). Hence, scores observed on k = 7 were always higher than that on k = 2, since k = 7 is nearer to k = 6 (Figure 5). Figure 4 shows results on simulated datasets for k = 6. We observed the following differences in stability behaviors among the four clustering algorithms.

• Different algorithms showed different stability behaviors until sample size reached n = 100. K-means showed high stability at smaller sample sizes as compared to the other methods.

• K-means, Fuzzy C-means and SOM showed fluctuation in scores even at large sample sizes, whereas CLARA showed consistent behavior (constant level of scores) at larger sample sizes.

• CLARA maintained 100% stability for larger sample sizes (300–500) whereas, SOM and Fuzzy C-means failed to reach 100% stability, even at large sample sizes. K-means showed stability scores between 0.7 and 1.0 most of the times for larger sample sizes.

Figure 4 suggests that K-means shows replicable performance than other non-hierarchical clustering algorithms considered (SOM, CLARA and Fuzzy C-means). Also, CLARA is a good choice for datasets of larger sample sizes.

## Discussion

We determined the performance of commonly used non-hierarchical clustering algorithms and the degree of stability achieved using several microarray datasets. We assessed cluster stability as a measure of replicability. We agree that replicability is not the only criteria for measuring cluster stability. However, a useful classification that characterizes some aspect of population must be replicable [2]. The most critical finding of this research was low stability achieved for all four clustering algorithms even at the elevated sample sizes of n = 50. This suggests that in general, given sample sizes up to 50, if the clustering algorithms we studied are applied, it is highly questionable that the results obtained will be meaningful. The extent to which these results apply to other clustering algorithms remains open to question, but we believe that the "burden of proof" is now on those who use clustering algorithms on microarray data and claim that such analysis produce replicable results.

Figure 3 and Figure 4 suggest that K-means shows replicable performance than other clustering algorithms considered (SOM, CLARA and Fuzzy C-means). K-means and SOM showed similar behavior in real datasets because they are closely related to each other. In K-means, centroids move freely in multidimensional space while they are constrained to a two-dimensional grid in SOM [28]. In SOM, the distance of each input from all reference vectors is considered, instead of just the closest one, weighted by the neighborhood kernel [29]. Thus, the SOM functions as conventional clustering algorithm if the width of the neighborhood kernel is zero [29]. Low stability achieved on all four clustering routines may also suggest that microarray datasets, in general, lack natural clustering structure. We do not claim that these results can predict the exact stability nature of a given dataset of a specific sample size, since these are generalized on a large number and variety of datasets. Nonetheless, the researcher should consider performing cluster analysis on large sample sizes to obtain more stable clustering solutions. Our research suggests a statistical criterion for selecting an appropriate number of clusters (k) for a given microarray dataset. This may be accomplished by computing Cramer's *v*^*2 *^on various values of k and selecting that value of k which provides a maximum stability score for a given dataset.

We also evaluated stability performances on simulated datasets. Simulated datasets helped us understand the stability behavior at large sample sizes (300–500). Datasets were structured for 6 clusters with a correlation of (0.33)^1/2 ^within clusters. All four clustering algorithms showed similar stability behavior in real and simulated datasets until sample sizes attained n = 50. K-means showed greater stability scores as compared to other methods at smaller sample sizes in both real and simulated datasets, indicating that K-means appear to be a better choice for datasets of smaller sample sizes. K-means and CLARA maintained 100% stability for large sample sizes (300–500), whereas SOM and Fuzzy C-means showed stability scores below 1, even at larger sample sizes (refer Figure 5).

Our methodology to compute stability used crisp assignments of genes to clusters. Hence, in Fuzzy C-means we assigned every gene to a cluster showing maximum degree of membership. We acknowledge that the above process of crisp assignment may affect the stability scores produced in Fuzzy C-means and hence expect it to produce low scores before hand. In SOM, we found that the choice of two-dimensional grid structure influences the stability scores produced on simulated datasets. For a same number of clusters (k) considered, we can create a two-dimensional grid in more than one way. Choosing the right grid structure for a given value of k to produce stable clustering solutions is beyond the scope of this paper and will address it in future investigations. Currently we limit the value of k (clusters) to 10; hence, if a real dataset has natural clustering structure for k greater than 10 (say k = 17), then this observation is not captured. We will consider measuring stability scores for higher values of k as an extension of this research. In conclusion our research suggests several plausible scenarios: (1) microarray datasets may lack natural clustering structure thereby producing low stability scores on all four methods; (2) the algorithms studied may not be well suited to producing reliable results and or (3) sample sizes typically used in microarray research may be too small to support derivation of reliable clustering results.

## Authors' contributions

NRG carried out statistical analysis and implementation of algorithms for cluster stability computation and wrote the first draft of the paper. DBA conceived of the study and participated in its design and coordination. GPP supervised the study and provided datasets for analysis. APS supervised the study and participated in its design. BSG gave constructive comments and suggestions on the design of the study. All authors read and approved the final manuscript.

## Figures and Tables

**Table 1:** List of microarray datasets considered for the study. Table 1 contains two columns of datasets. Each dataset is described by its name, source, and sample size (n). Table 1 shows 39 datasets. The first 3 columns list 19 datasets and last three columns describe 18 datasets.

**Name of the dataset**	**Source**	**Sample size (n)**	**Name of the dataset**	**Source**	**Sample size (n)**
GDS22	GEO	80	Leukemia dataset	[30]	70
GDS171	GEO	30	Medulloblastoma Data Set	[31]	34
GDS184	GEO	30	Prostate Cancer dataset	[32]	100
GDS232	GEO	46	Gaffney Head and Neck data	[33]	60
GDS274	GEO	80	Affymetrix Hu133A Latin Square	[34]	42
GDS285	GEO	20	CNGI design experiment	Unpublished	24
GDS365	GEO	66	Paired pre and post euglycaemic insulin clamp skeletal muscle biopsies	Unpublished	106
GDS465	GEO	90	GDS156	GEO	12
GDS331	GEO	70	GDS254	GEO	16
GDS534	GEO	74	GDS268	GEO	24
GDS565	GEO	48	GDS287	GEO	16
GDS427	GEO	24	GDS288	GEO	16
GDS402	GEO	12	GDS472	GEO	14
GDS356	GEO	14	GDS473	GEO	12
GDS389	GEO	16	GDS511	GEO	12
GDS388	GEO	18	GDS520	GEO	20
GDS352	GEO	12	GDS564	GEO	28
GDS531	GEO	172	GDS540	GEO	18
GDS535	GEO	12			

**Table 2:** List of simulated microarray datasets. Table 2 show the details of simulated datasets. Each of these datasets has clustering structure k = 6 (six clusters) with correlation *ρ *set to (0.33)^1/2^.

**Dataset Name**	**Sample size**	**Number of genes**	**Clusters**
Dataset1	20	1200	6
Dataset2	100	1200	6
Dataset3	200	1200	6
Dataset4	500	1200	6
Dataset5	1000	1200	6
Dataset6	40	1200	6
Dataset7	60	1200	6
Dataset8	80	1200	6

**Table 3:** Table showing stability results produced on a real dataset of sample size 16. Table 3 shows stability scores produced on a given dataset of a sample size of n = 16. We split the dataset into two halves each containing 8 subjects. The left dataset is resampled 6 times producing 6 samples of sample sizes 3 to 8, respectively. Similarly the right dataset is resampled to produce 6 samples. We measured the strength of the association between the clusters produced on every pair of samples (one sample from left and other from right dataset both of same sample size) using Cramer's *v*^*2 *^. Columns in the table represent number of clusters (k) and rows represent sample sizes. Stability score quantified for k = 10 and sample size 8 is 0.3699. This table shows there is 37% agreement between the clusters produced (k = 10) on pair of samples (a sample from left dataset and other from right dataset both of sample size 8).

K (CLUSTERS)										
		2	3	4	5	6	7	8	9	10

S A M P L E S I Z E	3	0.5883	0.47091	0.4503	0.4028	0.3809	0.3600	0.3313	0.3107	0.2992
	4	0.5799	0.48045	0.4244	0.3894	0.365	0.3469	0.3132	0.297	0.2858
	5	0.5738	0.48296	0.4297	0.3982	0.3644	0.3430	0.3195	0.3013	0.2790
	6	0.6433	0.54638	0.5142	0.4727	0.4405	0.4066	0.3817	0.3616	0.3396
	7	0.6534	0.54821	0.5250	0.4826	0.4462	0.4211	0.3915	0.3679	0.348
	8	0.6759	0.58447	0.5520	0.5045	0.4700	0.4592	0.4160	0.3975	0.3699
